# Tenofovir-Induced Renal Dysfunction Among HIV-Infected Patients: A Systematic Review

**DOI:** 10.7759/cureus.45787

**Published:** 2023-09-22

**Authors:** Yogamba M Shivakumar, Eshwar Burra, Kamran Shahid, Yonas Tamene, Shefali P Mody, Kaiser O Sadiq, Sai Sri Penumetcha

**Affiliations:** 1 Medicine, California Institute of Behavioral Neurosciences & Psychology, Fairfield, USA; 2 Internal Medicine, Government Medical College, Nizamabad, IND; 3 Internal Medicine/Family Medicine, California Institute of Behavioral Neurosciences & Psychology, Fairfield, USA; 4 Internal Medicine, California Institute of Behavioral Neurosciences & Psychology, Fairfield, USA; 5 Surgery, Plexus Neuro and Stem Cell Research Centre, Bangalore, IND; 6 General Medicine, California Institute of Behavioral Neurosciences & Psychology, Fairfield, USA; 7 General Medicine, Chalmeda Anand Rao Institute of Medical Sciences, Karimnagar, IND

**Keywords:** tenofovir alafenamide (taf), glomerular and tubular injury, kidney failure, hiv aids, tenofovir disoproxil fumarate (tdf)

## Abstract

Tenofovir disoproxil fumarate (TDF) is an antiretroviral drug widely used as part of antiretroviral therapy (ART) to treat human immunodeficiency virus (HIV-1) infection. Negative effects of tenofovir include impaired kidney function, especially with long-term use.

In studies conducted among HIV-positive individuals, we found evidence of extensive kidney damage associated with TDF use. Despite the therapeutic importance of this consequence, its continued use in ART regimens was not contraindicated. The therapeutic and long-term effects of TDF are a major concern. However, in countries or settings where resources are limited and renal function monitoring cannot be ensured, screening methods to detect ART-related renal failure are still supported by data. Therefore, it is safe to re-evaluate the use of TDF-based ART. However, adherence to guidelines may be hampered by insufficient laboratory testing in low- and middle-income countries. More research is also needed among people under 18 years of age and pregnant and breastfeeding mothers.

## Introduction and background

Tenofovir is one of the preferred backbone and essential components of highly active antiretroviral therapy (HAART) used in the management of human immunodeficiency virus (HIV-1) infection in both high-income and low-to-middle-income countries [1]. Tenofovir disoproxyl fumarate (TDF) and Tenofovir alafenamide (TAF) are both prodrugs of tenofovir, a potent nucleotide reverse transcriptase inhibitor. Both are converted intracellularly to the pharmacologically active moiety, tenofovir diphosphate [2]. Once activated, tenofovir acts with different mechanisms, including the inhibition of viral polymerase causing chain termination and the inhibition of viral synthesis [3].

Through a mix of glomerular filtration and proximal tubular secretion, tenofovir is eliminated unchanged in the urine. Organic anion transporters in the basolateral membrane (hOAT1, and to a lesser degree, OAT3) actively transport 20-30% of the drug into renal proximal tubule cells [3]. The apical membrane transporters multidrug resistance protein (MRP-4) and MRP-2 then release the drug into the tubular lumen. (Multidrug resistance proteins, encoded by ATP-binding cassette transporter sub-family C member (ABCC4) and ABCC2 genes [3,4]).

Tenofovir accumulates within cells because of increased entry from the human organic anion transporters (OAT1 and OAT3) and decreased efflux into the tubular lumen, which is the primary mechanism of TDF-induced nephrotoxicity [5]. TAF is a prodrug that absorbs more rapidly than TDF and causes cells to produce more tenofovir diphosphate, the drug’s active ingredient [5].

It is still unclear how the increased frequency of tubular injury in people living with HIV (PLWHIV) treated with TDF is caused. Apolipoprotein L1 (APOL1) is expressed in the proximal tubular cells of healthy kidneys and is responsible for nearly 70% of the excess risk of kidney disease in people of African ancestry [1]. Studies have shown that APOL1 genetic variants are strongly associated with HIV-associated nephropathy, a quickly progressing kidney disease with severe tubular damage. Around 87% of PLWHIV with chronic kidney disease (CKD) in Nigeria had two APOL1 risk alleles [4]. As a result, APOL1 renal risk alleles may contribute. Risk variants of APOL1 operate via a gain of injury or toxic activity on podocytes and other cells in the kidney, including proximal tubular epithelial cells [4].

According to a study conducted among more than 10,000 HIV-positive patients, the chance of developing CKD, proteinuria, and rapidly deteriorating kidney function increased by 34%, 11%, and 33%, respectively, for every year of exposure to TDF [5]. The rules of European societies recommend that patients using TDF with an estimated glomerular filtration rate (eGFR) of more than 60 mL/minute be given the option to switch medications if their eGFR has dropped more than 25% from baseline and/or has decreased by 5 mL/year for three or more years [5]. They also concur with the National Institutes of Health (NIH) guidelines that patients with baseline renal disease should use TDF cautiously or not at all [5].

According to multiple case reports and study reviews, tenofovir nephrotoxicity comprises Fanconi syndrome, proximal tubulopathy, or renal tubular acidosis [6-8]. Proteinuria, glucosuria, and hypophosphatemia, which result in osteomalacia [9] and proximal renal failure, are the hallmarks of Fanconi syndrome [8].

TDF-induced proximal renal tubulopathy is thought to be linked to high plasma concentrations and the subsequent accumulation of tenofovir in renal tubular cells [1]. This results in disrupting proximal tubular mitochondrial function. Acute tubular injury with abnormal mitochondrial ultrastructure and various degrees of tubule-interstitial scarring can be seen in kidney biopsies [1,6].

Risk factors for TDF-associated nephrotoxicity include advanced HIV illness, advanced age, low body weight, pre-existing renal impairment, concurrent use of ritonavir or cobicistat, the concomitant use of nephrotoxic drugs such as anti-tuberculosis (TB) medication in developing countries and having comorbidities [10,11]. Since the publication of the first instance in 2001 [11], observational studies have found more and more evidence of TDF’s renal toxicity. Data from reviews and meta-analyses showed that patients getting TDF-based therapies compared to non-TDF regimens had greater loss of kidney function and a higher risk of acute renal failure. However, low- and middle-income countries may have higher rates of renal problems in HIV patients due to a lack of renal function monitoring, inadequate management, restricted accessibility to dialysis and kidney transplant operations in case of renal failure, and other factors. Patients might not have the financial means to pay for both the costs of various treatments and renal function tests [10,12]. Many HIV-infected patients have been exposed to TDF and will continue to do so in the future, though TDF use is likely to decline over time, especially in resource-rich environments, with the introduction of TAF, another tenofovir prodrug with favorable kidney effects [10]. TDF is increasingly used in the treatment of HIV-1 infection (over nine million patient-years), particularly in settings with limited resources, despite mounting evidence of its association with nephrotoxicity.

Because of TAF’s improved plasma stability and higher intracellular accumulation of tenofovir diphosphate in target cells, antiviral activity has increased at lower doses while maintaining improved renal safety. Furthermore, there is sufficient from actual clinical contexts about whether patients who have cumulative TDF exposure are still at risk for glomerular tubular diseases even after stopping TDF.

The median duration to acquire renal tubular dysfunction on TDF is three to six months, and it is believed that this effect only manifests during the first 24 to 31 months of TDF use, which is why it is important to investigate the prevalence during this period.

## Review

Methodology

The Preferred Reporting Items for Systematic Reviews and Meta-Analyses (PRISMA) guidelines were followed for conducting this systematic review.

Data Sources and Study Design

We conducted a comprehensive search using PubMed and Google Scholar, starting on February 25, 2018, and ending on February 24, 2023. We incorporated data from primary research including cross-sectional studies, observational cohorts, case-control studies, and case reports documenting renal outcomes of HIV-positive patients on a TDF-containing regimen.

Eligibility Criteria

Studies that met the PICOS requirements, presented in Table 1 below, were considered for inclusion in this study.

**Table 1:** Inclusion criteria. ART: antiretroviral treatment; CrCl: creatinine clearance; eGFR: estimated glomerular filtration rate; HIV: human immunodeficiency virus; TDF: tenofovir disoproxil fumarate

Study design	Cohort, case-control, cross-sectional studies (if the duration of TDF therapy was stated)
Article characteristics	Full articles, open-access dissertations/theses
Study participants	Age group: more than or equal to 18 years. Males, females, and transgenders. Patients diagnosed as HIV positive and on TDF-containing ART
Intervention	TDF-containing ART
Outcome	Kidney dysfunction indicated by any of the following outcome measurements: beta 2 microglobulinuria, fractional excretion of phosphate, fractional excretion of uric acid, N-acetyl-b-D-glucosaminidase, non-diabetic glycosuria, glycosuria, phosphaturia, CrCl, eGFR, serum creatinine, albumin creatinine ratio

Exclusion Criteria

Articles not written in English, articles or original studies documenting the renal effects of TDF, and articles published before March 2018 were excluded. ART regimens without TDF or non-specific to TDF, such as indicators of renal impairment (in TDF-containing regimens), were excluded as well. In addition, patients on TDF but HIV-negative, pregnant and/or lactating women, and children (under 18 years of age) were excluded from the study. The MeSH keywords used in this study are presented in Table 2.

**Table 2:** Search keywords. HIV: human immunodeficiency virus; TDF: tenofovir disoproxil fumarate

Database	Keywords
PubMed	(((“Tenofovir”[MeSH]) AND “Tenofovir/adverse effects”[MeSH]) OR (“Kidney/drug effects”[MeSH] OR “Kidney/injuries”[MeSH])) OR “Antiretroviral Therapy, Highly Active/adverse effects”[MeSH]
Google Scholar	(“Tenofovir OR TDF”) AND (“renal dysfunction” OR “kidney dysfunction”) AND (“HIV seropositive”)

Data Abstraction

Three stages of article selection were used, namely, titles only, abstracts, and, finally, full-text articles. First, the titles and abstracts of the retrieved articles were examined to determine which research met the inclusion criteria.

Then, to establish which studies should be included in the qualitative analysis, the full texts of the chosen studies were retrieved and read.

Quality Assessment

The Newcastle-Ottawa checklist (for case-control and cohort studies) [13], Joanna Briggs Institute (JBI) quality appraisal checklist for case reports and cross-sectional studies [14], and PRISMA (for systematic reviews) were some of the tools used to assess the quality of the articles.

Results

**Figure 1:**
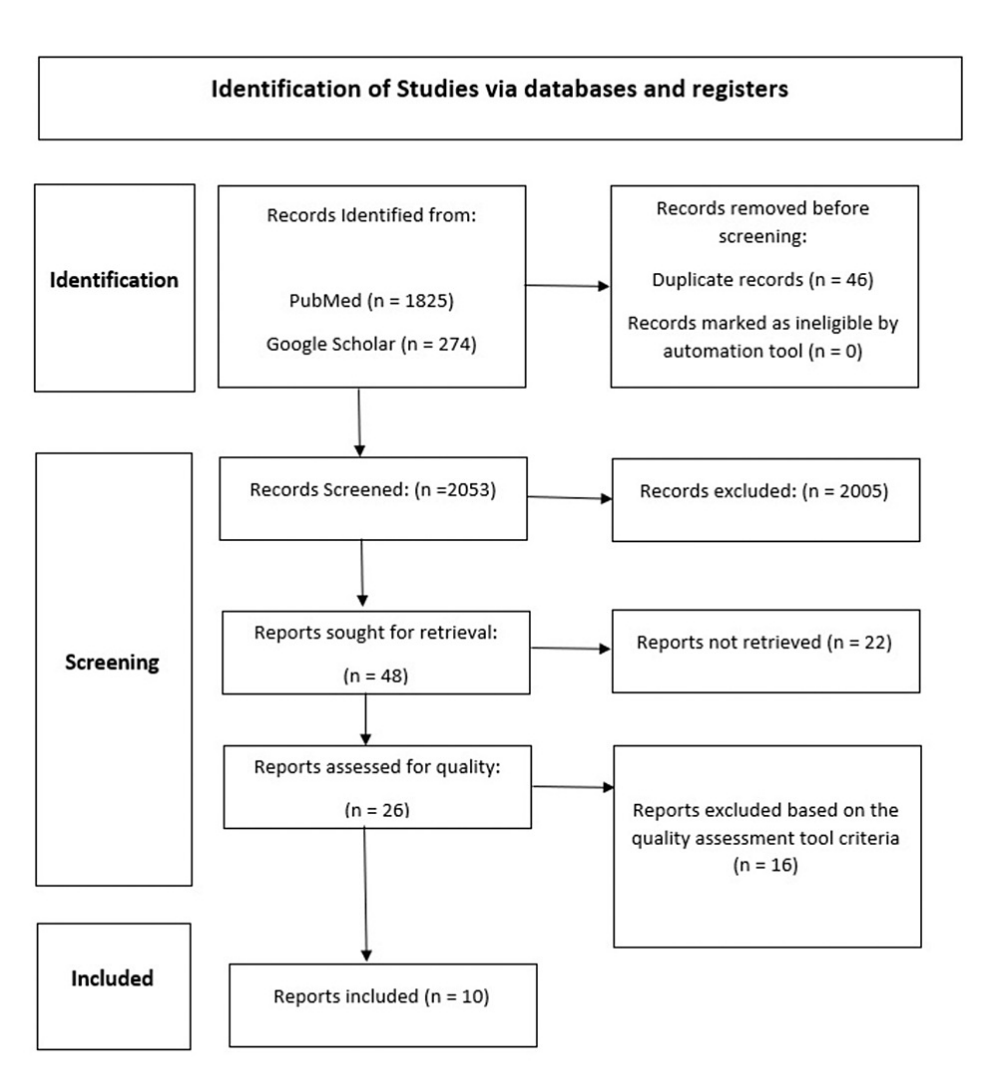
Preferred Reporting Items for Systematic Reviews and Meta-Analyses flowchart.

Only 46 of the 2,099 articles from two distinct databases that were returned by the internet search were duplicates, which were eliminated, leaving 2,053 items for screening utilizing the inclusion/exclusion standards listed in the tables above. In total, 2,005 articles were eliminated. Later, after retrieving 48 articles, 26 underwent a quality check. The search procedures are shown in Figure 1 below, and a total of 10 publications were chosen for the review and PRISMA checklist for the study design [15,16].

Study Variables

The sociodemographic features of the study participants (age, body mass index (BMI), weight, occupation, and educational attainment), drug-related factors (TDF and non-TDF ART regimens, concurrent use of ART, anti-TB medicine, cotrimoxazole, and concurrent use of a nephrotoxic drug), and drug-related factors were all independent variables.

HIV-related factors include viral load, CD4, WHO stages one through four, opportunistic infections including TB and *Pneumocystis carinii* pneumonia, as well as concomitant conditions such as diabetes, cancer, and high blood pressure. Renal dysfunction was the study’s dependent variable, though. Table 3 lists the demographic and clinical parameters of each study.

**Table 3:** Demographic and clinical parameters of each study. ABC: abacavir; ART: antiretroviral treatment; BMI: body mass index; CrCl: creatinine clearance;  CD4: cluster of differentiation 4; eGFR: estimated glomerular filtration rate; IQR: interquartile range; MSM: men who have sex with men; PVL: plasma viral load; TDF: tenofovir disoproxil fumarate

Author and year of publication	Predominant sex	Weight/BMI	Age	WHO clinical Stage	Viral copies/mL	CD4 cells/mm^3^
Yazie et al. 2019 [17]	Sample size = 63. Females = 68.3%	BMI = 22.6 kg/m^2^	Mean age = 37.5 years	Stage 1	-	241 (IQR = 106–457)/mm^3^
Agrawal et al. 2022 [18]	Sample size = 100. Males = 50% and females = 50% (equal)	Weight = 56–105 kg	Mean age = 38.75 years	-	-	500–1,500 cells/mm^3^
Lee et al. 2019 [19]	Sample size = 108. Male = 86.1%	Weight = 63.48 to 83.48 kg. BMI – 22.1 ± 3.57 kg/m^2^	Mean age = 42 years	-	TDF vs. ABC, 3.95 ± 1.82 log10/mL vs. 3.53 ± 2.25 log10/mL (p = 0.137)	TDF vs. ABC, 318.19 ± 231.81/μL vs. 312.94 ± 267.33/μL (p = 0.879)
Agbaji et al. 2019 [20]	Sample size = 4,897. Females = 61%	Weight = 61 to 70 kg	Mean age = 42 years	Stage 3 or 4	Less than or equal to 10,000 copies/mL	>100 cells/mm3 (72%) prior to ART initiation
Tan et al. 2019 [21]	Sample size = 269. Males = 97.0%	Weight = 62 kg. BMI = 20.9 kg/m^2^	Mean age = 29 years	-	36,000 copies/ml (IQR = 12,600–102,000)	312 cells/mL (IQR = 234–402), PVL 36,000 copies/mL (IQR = 12,600–102,000)
Hsu et al. 2020 [22]	Sample size = 4,743. Females = 12%	Weight = 55 to 63 kg. BMI = 25–26 kg/m^2^	Low-risk group: median age 29–31 years. Medium and high-risk group: median age: 49–52 years	-	-	243–344 cells/μL
Yilma et al. 2020 [23]	Sample size = 225. Females = 79.2%	Weight - 44.1 to 54.3 kg. BMI > 16 kg/m^2^	Mean age = 45 years	Stage 3 or 4 disease	-	200 cells/mm^3^ to <350 cells/mm^3^ and WHO stage 3 or 4 disease
Karoney et al. 2022 [24]	Sample size = 516. Females = 60.3%	Weight = 58.0 to 63.0 kg. BMI = 23.0 kg/m^2^	Median age: 41.5 to 63.1 years	Stage 3 (TDF 40% vs. non-TDF 34.5%; p = 0.02)	Undetectable viral load	TDF 323 vs. Non TDF 370 cells/mm^3^; p = 0.05
Ng'umbi et al. 2022 [25]	Sample size = 145. Females = 57.9%	Weight = 44 to 54 kg. BMI = 18.6 kg/m^2^	Median age = 46 to 54 years	-	Undetectable viral loads with a mean of 69.7 (± 45.9)	Median CD4 227c/μl (IQR = 128–335) at the time of TDF initiation
Nishijima et al. 2018 [26]	Sample size = 941. Males = 94%	Weight = 60.2 to 76.3 kg. BMI = 23.5 kg/m^2^	Mean age = 45 years	-	Less than 50 copies/mL	575 cells/mL

Characteristics of Included Studies

Of the 10 articles considered for this analysis, three were cohort studies, three were cross-sectional studies, one was a non-randomized cross-sectional study, one was a prospective observational study, and two were retrospective cohort studies. Sample sizes varied from 60 to 9,802. The length of time on tenofovir-containing ART ranged from six months to seven to ten years.

Although TDF was a part of every study’s ART regimen, different ART combinations, concomitant medicines, and their durations were used in different research. Not all studies included information on comorbidities or concomitant drugs. Most trials enrolled participants who had been TDF users for at least six months. Table 4 lists the characteristics of each study.

**Table 4:** Study characteristics. ABC: abacavir; ART: antiretroviral treatment; CKD: chronic kidney disease; DAD: data collection on adverse events of anti-HIV drugs; NNRTI: non-nucleoside reverse transcriptase inhibitor; NRTI: nucleoside reverse transcriptase inhibitor; PI: protease inhibitor; TDF: tenofovir disoproxil fumarate; ZLA: zidovudine lamivudine abacavir; ZLN: zidovudine lamivudine nevirapine; ZLE: zidovudine lamivudine efavirenz

Author and year of publication	Country	Study type	Sample size (TDF-containing regimen)	Comparison group	Baseline duration on ART	Follow-up duration
Yazie et al. 2019 [17]	Ethiopia	Prospective observational study	63 TDF + lamivudine + efavirenz OR TDF + lamivudine + ritonavir boosted atanzanavir	-	-	6 months
Agrawal et al. 2022 [18]	India	Non-randomized cross-sectional study	50 TDF regime (tenofovir/lamivudine/efavirenz)	50 non-TDF (ZLN, ZLA, ZLE)	At least 1 year. (1 to 7 years)	-
Lee et al. 2019 [19]	Korea	Single-center retrospective cohort	108 TDF-based regimen	102 were ABC-based ART group	-	For ABC-based ART group 109.01 ± 26.96 weeks. For 79.68 ± 36.39 weeks for TDF
Agbaji et al. 2019 [20]	Nigeria	Cohort	TDF-exposed patients: 2,104	TDF-unexposed patients: 2,793	Naive at ART initiation	144 weeks
Tan et al. 2019 [21]	China	Single-center retrospective observational study	269 patients on TDF + lamivudine +efavirenz	-	ART naïve	baseline, 12, 24, 48, 96, 144, and168 weeks
Hsu et al. 2020 [22]	USA	Cohort	6,222 TDF-based regimen. Low risk: 4,743. Medium risk: 994. High risk: 484. The first composite exposure of interest consisted of TDF use (yes/no) and baseline DAD CKD risk group (low, medium, and high-risk). The second composite exposure consisted of TDF use (yes/no) and whether the ART regimen included a pharmacoenhancer (cobicistat or ritonavir, yes/no) standard ART regimen (two NRTIs and one core agent: non-boosted integrase strand transfer inhibitor, non-boosted nonnucleoside reverse transcriptase inhibitor (NNRTI), boosted elvitegravir, or boosted PI)	3,580 non-TDF-based regimen. Low risk: 2,827. Medium risk: 481. High risk: 272. The no-TDF/low-risk group served as the referent group in comparative analyses. The no-TDF/non-boosted ART regimen group served as the referent group. in comparative analyses	-	24,382 person-years
Yilma et al. 2020 [23]	Ethiopia	Cohort	Naïve TDF, NNRTI regimen: 279	Non-TDF, NNRTI regimen: 60	-	12 months
Karoney et al. 2022 [24]	Western Kenya	Cross-sectional study	TDF group: 261	Non-TDF group: 255	Median duration of ART: 6 years	-
Ng'umbi et al. 2022 [25]	Uganda	Hospital-based, cross-sectional study	TDF-based regimen: 145	-	24 ± 2 months	Cross-sectional study
Nishijima et al. 2018 [26]	Japan	Prospective cross-sectional study	Current TDF users: 371. Past TDF users: 233	Never TDF users: 337	6 to 10 years	Cross-sectional study

Guidelines to Monitor Renal Function

No randomized controlled trials have evaluated the best kind and frequency of monitoring yet. The data are based on short-term randomized clinical trials of ART techniques, long-term epidemiology data, and observational cohort data. However, to establish an evidence-based strategy for routine laboratory monitoring of antiretroviral toxicity, a guideline was created by the New York State Department of Health’s (NYSDOH) Acquired Immune Deficiency Syndrome (AIDS) Institute.

The NYSDOH advises patients under 50 without chronic comorbidities to have a minimum laboratory monitoring frequency with the start or change of ART. eGFR at the start of treatment, three and 12 months after the start of TDF, and annually after that. Additionally, test for proteinuria (by urine analysis or protein-to-creatinine ratio), glucosuria, and serum phosphorus at baseline, 12 months after the start of TDF, and yearly after that. However, in countries with few resources, it is nearly impossible to implement.

Renal Outcome Parameters

Most studies used eGFR as a measure of renal outcomes (using one or a combination of the Cockcroft-Gault, modification of diet in renal disease, and chronic kidney disease epidemiology collaboration (CKI-EPI) equations). Only a small number of studies indicated at least creatinine clearance (CrCl) as the result. Studies that aim to assess tubular injury caused by TDF are defined by a collection of parameters including plasma bicarbonate levels, beta 2 microglobinuria, glucosuria, and fractional excretion of phosphate and uric acid. Table 5 lists the definitions of renal outcomes between studies.

**Table 5:** Study findings and conclusions of each study. ABC: abacavir; AOR: adjusted odds ratio; ART: antiretroviral treatment; CI: confidence interval; CD4: cluster of differentiation 4; CKD: chronic kidney disease; DAD: data collection on adverse events of anti-HIV drugs; eGFR: estimated glomerular filtration rate; HIV: human immunodeficiency virus; IQR: inter quartile range; KTD: kidney tubular disease; NNRTI: non-nucleoside reverse transcriptase inhibitor; OR: odds ratio; PLWH: people living with HIV; PTRD: proximal tubular renal dysfunction; TDF: tenofovir disoproxil fumarate; TLE: TDF lamivudine efavirenz

Author and year of publication	Results	Conclusion about TDF relation
Yazie et al. 2019 [17]	16 (25.4%) of the 63 research participants showed an eGFR decline of more than 25% from baseline. The eGFR had changed on average by -8.4 mL/minute/1.73m^2^ from the beginning of the trial after six months. CKD was diagnosed in 2 (3.2%) of the study participants, and it was validated by two consecutive measurements of eGFR less than 60 mL/minute/1.73m^2^ at a 4-month interval. The prevalence of proteinuria at the end of six months was higher than the prevalence of proteinuria at baseline (27% and 20.6%, respectively). 14.3% of the 27% of proteinuria patients who had renal impairment also had proteinuria. However, the prevalence of glycosuria at the end of six months remained constant with the baseline frequency	Renal impairment was identified in one-fourth of the individuals in this trial, raising concerns about the long-term use of TDF
Agrawal et al. 2022 [18]	Serum creatinine levels at baseline and during the study period indicated a positive correlation (0.233) with a significant correlation and dependence (0.020) for all 100 patients when comparing the TLE with the non-TLE-based regimens. Baseline serum creatinine and at the time of the study were somewhat correlated (0.574) for the TLE regimen, and the association was both significant (0.000) and dependent (0.001). When compared to the non-TLE regimen, the relationship was positively correlated (0.375), the correlation was significant (0.007), but the reliance was not (0.067). Similarly, there was a substantial positive correlation between hemoglobin levels at the start of the trial and at the end, which is significant (0.710), and there was a significant (0.000) reliance. When serum uric acid from the TLE group was compared to the non-TLE group, it was discovered that there was a negative connection (-0.220), and the correlation was not significant (0.125)	Patients who had been exposed to the drug for at least a year reported experiencing nephrotoxicity. A risk factor for nephrotoxicity was prolonged exposure to the TLE regimen
Lee et al.. 2019 [19]	The trial comprised 210 HIV-positive patients in total, of whom 108 received TDF-based ART and 102 received ABC-based ART. In the TDF group, 16 patients (14.8%) and in the ABC group, 11, both experienced renal dysfunctions. The prevalence of renal dysfunction was 9.66 per 100 person-years (PYs) in the TDF group and 5.14 per 100 PYs in the ABC group, respectively (p = 0.176). Renal dysfunction rates in the propensity-score matched analyses were TDF 13.3% versus ABC 13.3% (p > 0.999)	In Korean patients with human immunodeficiency virus infection, tenofovir disoproxil fumarate usage was not linked to nephrotoxicity if baseline renal function was normal
Agbaji et al. 2019 [20]	Data from 4,897 patients were evaluated, with a median age of 42 years (interquartile range: 36–49) and 61% females. In subjects exposed to TDF, the prevalence of renal impairment increased from 10% at week 24 to 45% at 144 weeks, while in participants not exposed to TDF, the prevalence increased from 8% at week 24 to 14% at 144 weeks. At 144 weeks of ART, exposure to tenofovir disoproxil fumarate predicted a risk of renal impairment (odds ratio: 2.36; 95% confidence interval: 1.28–4.34)	Renal impairment is considerably more likely to occur after prolonged TDF-based ART treatment. It is important to examine whether TDF-based treatment is still appropriate in our setting
Tan et al. 2019 [21]	97.0% of the study participants were men, with a median age of 29 and an eGFR of 124.0 mL/minute/1.73m^2^. Renal impairment occurred in 7 (2.7%) patients following 168 weeks of ART. When compared to week 12 observation, the incidence of diminished renal function was substantially higher at week 168 (24.8% vs. 3.7%)	In Chinese HIV-positive patients with a median age of 29 years and no comorbidities, TDF-related renal impairment remained uncommon. Renal function was linked with lower weight and shorter ART duration
Hsu et al. 2020 [22]	All participants had at least one eGFR measurement prior to the start of ART, with the most recent reading being below 60 mL/minute/1.73m^2^ within the previous 12 months or at the time of ART commencement, and at least two eGFR measurements following the start of ART, with at least 90 days between the first and last follow-up eGFR measurements. 24.382 person-years of follow-up revealed a total of 125 CKD occurrences. The IRs were comparable across TDF exposure strata within the DAD CKD score stratum, with the highest incidence being correlated with the highest baseline CKD risk. There was no statistically significant difference between the low-risk group with TDF and the low-risk group without TDF regarding the chances of incident CKD (adjusted odds ratio = 0.55, 95% confidence interval: 0.19-1.54). By using either TDF or pharmacoenhancer exposure, the odds of incidence. CKD did not statistically differ considerably	After the start of ART, incident CKD was uncommon and strongly associated with baseline CKD risk. In the biggest group of ART-naive PLWH, those with a low baseline DAD CKD risk, TDF-containing regimens did not increase the odds of CKD and may still be an effective therapeutic choice in the right settings
Yilma et al. 2020 [23]	With 12 months of ART, the median (IQR) eGFR change was 0.8 (-11.1; 10.0) mL/minute/1.73m^2^. At 12 months, the eGFR of about 41 and 26.9% of HIV patients had decreased by more than 3 and 10 mL/minute/1.73m^2^, respectively. However, none of the HIV patients had a drop to a level below 60 ml/minute/1.73m^2^ over a year. Additionally, none of the HIV individuals developed persistent proteinuria or glycosuria. The eGFR was significantly lower at 12 months after the start of ART in older HIV patients, particularly those over 45 years old and those whose viral loads were not controlled after 6 months of ART. However, there was no difference in 12-month eGFR between HIV patients who started on a TDF-based regimen and those who did not	Over a 12-month period, renal function was stable in HIV patients receiving either a TDF- or non-TDF-based NNRTI-based ART regimen
Karoney et al. 2022 [24]	In the TDF group, the proportion of PTRD was 10.0%, compared to 3.1% in the group that did not use TDF (p = 0.001). The mean eGFR for the TDF group compared to the TDF-sparing group was 112.8 (21.5) vs. 109.7 (21.9) mL/minute/1.73m^2^ (p = 0.20). AOR: 3.0. TDF users were more likely to have PTRD than non-TDF users (CI = 1.12 to 7.75). In the two groups, the median amount of time using ART was 6 years. Metabolic acidosis (41.8 vs. 35.7%) and tubular proteinuria (18.8% vs. 6.3%) were more prevalent in TDF users than in TDF-sparing regimens	When compared to the TDF-sparing group, the TDF had a substantial impact on PTRD, although eGFR was not significantly different
Ng'umbi et al. 2022 [25]	In all, 116/145 (80%) subjects had at least one abnormality in the parameters of tubular dysfunction; 26 (17.9%) and 3 (2.1%) of the participants, respectively, exhibited a combination of two or more parameters. 20% (29/145) of the participants in our study had renal tubular dysfunction (95% CI = 14.2-27.3)	Despite a normal serum creatinine level, renal tubular dysfunction is very common and can happen in people using TDF. To identify renal tubular failure, we advise using urine abnormalities as opposed to isolated serum creatinine
Nishijima et al. 2018 [26]	With a median age of 45, an estimated glomerular filtration rate of 75 ml/minute/1.73m^2^, and a CD4 count of 575 cells/mL, 94% of the research participants were men. Nearly 98% were on antiretroviral medication. 39% of patients presently take TDF, and 64% of patients have ever used TDF. TDF was used by 29% of people for more than 5 years. 116 (12%) patients had KTD diagnosed. More than 5 years of TDF exposure and current TDF use were significantly associated with KTD in the multivariate model, as were more than 5 years and past TDF use (OR 2.4, 95% CI = 1.09–5.33), less than 5 years and current TDF (OR = 2.4, 95% CI = 1.24–4.85), and less than 5 years and never TDF use (OR = 2.4, 95% CI = 1.22–4.64). Using 4 or 3 years of exposure as the cutoff had the same effects. Less than 2 years and current TDF (OR = 2.3, 95% CI = 0.84-6.20) and Less Than 2 Years and past TDF (OR = 1.9, 95% CI = 0.73–4.93), however, were not associated with KTD with 2 years of exposure, but more than 2 years and both current and past TDF were	KTD and cumulative TDF use were strongly and robustly associated. According to the study’s findings, if patients used TDF for more than two years, their KTD may continue even after TDF is stopped

Discussion

The purpose of this review was to describe the various effects of renal dysfunction caused by TDF in adult PLHIV-infected patients on ART as well as the risk factors for renal dysfunction. A total of 17,282 people across 10 studies in nine countries were included in the studies, ranging in sample size from 60 to 9,802. Table 6 lists the objectives and definitions of renal dysfunction for each study.

**Table 6:** Objectives and definitions of renal dysfunction of each study. ADR: adverse drug reaction; CKD: chronic kidney disease; CKD-EPI: chronic kidney disease epidemiology collaboration; DAD: data collection on adverse events of anti-HIV drugs; eGFR: estimated glomerular filtration rate; FE: fractional excretion; HIV: human immunodeficiency virus; KTD: kidney tubular disease; MDRD: modification of diet in renal disease; PLWH: people living with HIV; sCr: serum creatinine; TDF: tenofovir disoproxil fumarate; TLE: TDF lamivudine efavirenz; WHO: World Health Organization

Author and year of publication	Age group of interest	Definition	Objectives
Yazie et al. 2019 [17]	≥18 years	After starting a TDF-based antiviral therapy, renal dysfunction was defined as an eGFR drop of more than 25% from baseline. In patients with HIV, eGFR was calculated using the CKD-EPI equation	To evaluate the prevalence of renal impairment, risk factors for it, and the average change in eGFR in HIV-infected patients taking TDF-based antiretroviral therapy. This study serves as a guide for the early identification of renal impairment
Agrawal et al. 2022 [18]	18–60 years	If the following conditions were satisfied, nephrotoxicity was determined: 1. An increase in serum creatinine of 0.3 mg/dL within two days, 1.5 to 1.9 times baseline within seven days, or 0.3 mg/dL but within normal limits is also a sign of significant renal damage because the usual range is between 0.5 and 1.5 mg/dL. 2. Hypouricemia as observed in Fanconi syndrome affects the proximal tubules (normal values: 2.6–6.0 mg/dL). 3. Unusual grades of +1 and +2 for the albumin-creatinine ratio in spot urine. 4. Anemia (normal hemoglobin for women is 12 and for men is 13)	The purpose of this study was to (1) Determine the frequency of nephrotoxicity brought on by tenofovir in the TLE regimen and compare it to non-tenofovir-based antiretroviral regimens using laboratory criteria. 2. To determine the time frame for the beginning of nephrotoxicity. 3. To analyze the causality of ADR using the WHO causality scale
Lee et al. 2019 [19]	More than 20 years	Greater than a 25% reduction in the initial eGFR was considered renal impairment. Using the CKD-EPI equation, eGFR was calculated	The study performed a single-center retrospective cohort to assess the prevalence and contributing variables of TDF-associated nephrotoxicity in HIV-infected patients in Korea
Agbaji et al. 2019 [20]	>18 years	Renal impairment was deemed to exist when at least one of two criteria was met. According to the Kidney Disease Outcomes Quality Initiative recommendations, stage 3 or greater renal disease is defined as eGFR less than 60 mL/minute/1.73m^2^ (calculated using the (MDRD) calculation). The risk, injury, failure, loss, and end-stage kidney criteria define renal injury as a rise in SCr higher than or equal to two times that of baseline or an increase in CrCl greater than 50% from baseline on one or more measurements	Study to assess the impact of prolonged TDF exposure on renal function in a cohort of HIV-1-infected Nigerians
Tan et al. 2019 [21]	>18 years	eGFR less than 90 mL/minute/1.73m^2^ or a reduction of more than 25% from baseline were both considered signs of TDF-related renal impairment. For the CKD-EPI, a reduction in eGFR of more than 10 mL/minute/1.73m^2^ from baseline was considered to indicate decreased renal function. B2 microglobulin’s normal range was less than 0.5 mg	Assessed the prevalence of renal impairment among Chinese patients who had received TDF-containing ART regimens for an extended period
Hsu et al. 2020 [22]		eGFR less than 60 mL/minute/1.73m^2^, more than two instances in a 90-day period apart, was considered to indicate CKD. Using the DAD CKD risk score, the baseline risk of developing CKD for each individual was calculated	A large cohort study of PLWH in the USA. Using the baseline DAD CKD risk score, determine the risk of CKD related to the use of TDF. The goal of this study was to comprehend how baseline CKD risk affected this association. Pharmacoenhancers’ effects on the discovered link between TDF and CKD were also assessed
Yilma et al. 2020 [23]	≥18 years	The participants were divided into three groups based on the change in eGFR over the previous 12 months: “decliner” if the change was >3 mL/minute/1.73m^2^, “stable” if the change was between -3 and 3 mL/minute/1.73m^2^, and “riser” if the change was >3 mL/minute/1.73m^2^	Focus on TDF, sought to evaluate parameters related to renal function changes throughout the first year of ART
Karoney et al. 2022 [24]	18–79 years	Any two of the following four parameters—metabolic acidosis, beta-2 microglobulinuria, and fractional excretion of phosphate greater than 20%—were considered indicators of proximal tubular renal failure. Normoglycemic glucosuria was characterized by dipstick detection of glucose in the urine despite a random blood glucose of less than 11.1 mmol/L. Less than 20 mmol/L of plasma bicarbonate was considered metabolic acidosis. Beta 2 microglobulin levels in urine that are high (more than 0.3 mg/mmol) are referred to as tubular proteinuria. Fractional excretion of phosphate (FEphos) of more than 20% in persons with normal blood phosphate levels (0.85 to 1.45 mmol/L) or more than 10% in participants with hypophosphatemia (serum phosphates of less than 0.85 mmol/L)	This cross-sectional study evaluated the overall renal function, proximal tubular renal impairment, and their predictors among patients on TDF-containing versus TDF-sparing regimens
Ng'umbi et al. 2022 [25]	>18 years	When at least two of the following criteria are present, it is renal tubular dysfunction. Phosphate wasting: FEphos levels of more than 20% in individuals with normal serum phosphate levels (2.7–4.5 mg/dL or 0.87–1.45 mmol/L) or more than 10% in patients with hypophosphatemia (serum phosphate levels of less than 2.7 mg/dL or 0.87 mmol/L) Glucosuria: urine glucose greater than 1.7 mmol/L with plasma glucose less than 10 mmol/L; uric acid wasting: FE of uric acid greater than 10% in presence of decreasing plasma uric acid level (men less than 0.20 mmol/L, women less than 0.15 mmol/L)	The purpose of the study was to identify the prevalence and risk factors for renal tubular dysfunction in Ugandan HIV-positive individuals taking TDF-containing regimens
Nishijima et al. 2018 [26]	20 years and above	The presence of two or more abnormalities in any of the five tubular markers—beta 2 microglobulinuria, fractional excretion of phosphate, fractional excretion of uric acid, N-acetyl-b-D-glucosaminidase, or nondiabetic glycosuria—was predefined as KTD. The Japanese equation created by the Japanese Society of Nephrology was used to determine eGFR based on standardized serum creatinine, sex, and age	This research was done to show that TDF increases the risk of renal tubular dysfunction and to determine if kidney tubular dysfunction (KTD) endures after TDF is stopped

Yazie et al. [17] found a positive association between TDF exposure and renal outcome. However, only baseline proteinuria, baseline CD4 count less than 200 cells/mm^3^, and age >50 years were substantially linked to renal impairment. Moreover, the bulk of these cases occurred in the first month. CKD was identified in two (3.2%) study participants and was validated by two consecutive measurements of eGFR 60 mL/minute/1.73m^2^. The prevalence of APOL1 risk mutations for renal illness was shown to be low among Ethiopians compared to other Africans, even though CKD assessment was not the study’s primary goal. The long-term usage of TDF is questioned in this study because one-fourth of the individuals had renal failure diagnosed. The small sample size and short follow-up period are limitations of this study. Renal dysfunction linked with tenofovir may also be understated because renal tubular dysfunction was not examined in this investigation.

In contrast to other studies, Agrawal et al. [18] found that patients who experienced nephrotoxicity were not underweight, and age was not a factor either, unlike in earlier studies where advanced age was an independent risk factor. They never discovered an association between the mean CD4 count of patients in both groups and the development of nephrotoxicity (500-1,500 cells/mm^3^) because all the patients in both groups had below-normal CD4 counts. Because serum creatinine would not exceed the normal limit until GFR is 63/mL/minute/1.73m^2^, there is a possibility that the incidence of nephrotoxicity was underestimated. Patients who received the medication for at least a year reported nephrotoxicity, with a longer time under the tenofovir lamivudine efavirenz regimen serving as a predisposing factor. While serum creatinine was elevated by 0.3% in the remaining 12 (24%) patients taking tenofovir, none of them had hypouricemia, anemia, or an abnormal urine albumin-to-creatinine ratio, indicating that serum creatinine may be the first parameter to become abnormal in renal affection and necessitates intervention to avoid the progression of nephrotoxicity. The study suggested considering switching to an abacavir (ABC)-based regimen or using TAF.

Lee et al. [19] reported that, in HIV-positive Korean patients with baseline normal renal function, TDF usage was not related to nephrotoxicity, and an independent risk factor associated with nephrotoxicity was identified as category C from the Centers for Disease Control and Prevention.

Due to the substantially higher exclusion rate of patients with serious conditions (category C) in the TDF-based ART group compared to the abacavir (ABC)-based ART group, the difference in the rates of renal dysfunction between the two groups was reduced. There was a difference in the baseline eGFR between the two groups. This discrepancy may have been caused by the fact that physicians frequently recommended ABC-based ART rather than TDF-based ART to patients with lower renal function or several risk factors for renal failure. TDF-based ART was more frequently used in patients with HIV and HBV co-infections than ABC-based ART because the anti-retroviral drug tenofovir, which is effective against both HIV and HBV, can also prevent the development of clinically significant liver disease by directly inhibiting HBV replication. Due to the timing gap between the introduction of ABC (in 2002) and TDF (in 2012), the ABC-based ART was used for a longer period than the ABC-based ART.

A cohort conducted by Agbaji et al. [20] found that prolonged exposure to TDF increases the likelihood of renal function decrease. The percentage of patients with renal impairment among TDF-exposed patients increased from 13% at 48 weeks to 35% and 45% at 96 and 144 weeks of ART, respectively, in contrast to the 14% renal impairment at 144 weeks found among patients who were not exposed to TDF. Proteinuria was not frequently tested for at the start of ART in the earlier years because it was not advised by recommendations; therefore, researchers were unable to assess its impact on the study’s findings. The most crucial element in the onset and development of CKD is proteinuria. Due to the study population’s predominance of young adults, there was only a weak correlation between older age and renal impairment. At 96 and 144 weeks of ART, there were significantly more patients with hepatitis B or C coinfection than those who were not (21% against 15% and 28% versus 19%, respectively) who had renal impairment. At 144 weeks of ART, renal impairment was linked to the WHO clinical stage three or four and a CD count of less than 100 cells/mm^3^ before treatment. Additionally, baseline eGFR was much higher in individuals with renal impairment compared to those without renal impairment (median (interquartile range) of 96 (75-120) versus 92 (71-114) mL/minute/1.73m^2^). According to Nigerian HIV treatment recommendations, the first ART regimen for adults and adolescents who use TDF-based ART should be reviewed, especially in environments where renal function monitoring cannot be ensured. Long-term use of TDF-based ART poses serious renal safety issues with HIV-1 patients. The author urges Nigeria and other resource-constrained nations to swiftly adopt a tenofovir alafenamide-based regimen as part of their HIV treatment guidelines.

Tan et al. [21] study did show that exposure to TDF for periods longer than 144 weeks was linked to decreased renal function, highlighting the need for studies with longer follow-up periods and regular monitoring of renal function in older patients with comorbidities during the treatment phase given the effect of long-term exposure to TDF on renal function. Because young patients made up the majority of the study’s participants, the findings might not apply to women, the elderly, or patients with concomitant conditions. Patients who weighed less than 58 kg were 2.23 times more likely than patients who weighed more than 67 kg to experience an eGFR drop of greater than 10 mL/minute/1.73m^2^ following 168 weeks of ART. Even though there was only one patient with renal impairment, the study found that high beta 2 microglobulinuria was negatively correlated with eGFR, which suggests that TDF produced more renal tubular injury than was clinically apparent from changes in eGFR.

Hsu et al. [22] reported that people with high baseline D:A:D CKD risk scores, regardless of TDF use, had a higher chance of developing CKD than those with low baseline risk scores and no TDF use. Considering these findings, the researcher draws the conclusion that TDF may still be considered the first line of treatment for low-risk patients in circumstances when doing so would improve access to first-line medications. This strategy, however, would necessitate the accurate identification of those with low baseline CKD risk before the start of ART, as well as the appropriate review of kidney function and CKD risk throughout the course of therapy. Even though past studies have indicated that TDF toxicity rises with exposure time, cumulative TDF exposure was not evaluated in this study. The lesser effect of TDF and pharmacoenhancers may be partially explained by this.

Yilma et al. [23], with a focus on TDF during the first year, at 12 months after starting ART, eGFR was lower among older HIV-positive adults and people whose viral loads were unsuppressed at six months. The study did not discover any differences in 12-month eGFR between-based versus non-TDF regimens. In persons with HIV who had not had ART, they discovered that a higher CRP was linked to a reduced eGFR. Because age and muscle mass are considered in eGFR calculations to estimate renal function, it is possible that CRP has a stronger correlation with eGFR than serum creatinine. It was difficult to compare research participants to individuals with greater CD4 levels as they had severe immunosuppression and/or advanced HIV illness. At the time of the start of ART, the HIV population in this cohort was younger and had normal kidney function. Less common traditional risk factors (rate of co-infection with hepatitis B and C) were present in the participants, and they did not take other antiretroviral medications such as protease inhibitors. The nutritional intervention may also affect the way blood creatinine is metabolized and may have an impact on changes in eGFR as the study was primarily designed to test nutritional interventions. In addition, the comparatively normal kidney function at the start of ART may be due to the absence of APOL1 genetic variations in people of Ethiopian origin, which lowers Ethiopians’ vulnerability to HIV.

In a cross-sectional study, Karoney, et al. [24] indicated that HIV patients on TDF had significantly more proximal tubulopathy than those on a TDF-sparing regimen. The mean eGFR in the two groups did not differ significantly. As ART use in the two groups was used for an average of six years, these results could change over a longer period. TDF use and a normal BMI, which were determined to be protective, were linked to proximal tubular renal dysfunction PTRD. A priori chosen as risk variables, additional characteristics such as age, sex, length of ART usage, viral load, and presence of comorbidities were not substantially linked to PTRD. According to the researcher, patients receiving TDF should be advised to undergo routine testing of tubular function parameters.

A hospital-based, cross-sectional study was conducted in Uganda by Ng'umbi et al [25] in PLHIV on TDF-containing regimens. This study demonstrated the existence of Fanconi syndrome parameters, which is consistent with TDF-associated renal tubular dysfunction. Unlike other studies where various definitions were employed, researchers used the set of criteria that were used to define Fanconi syndrome. After controlling for age, the study found a significant relationship between gender and renal tubular disease. Renal tubular dysfunction was linked to the usage of protease inhibitors concurrently and for longer than two months. Researchers speculated that this finding may be caused by polymorphisms in the genes encoding drug transporters, but they were unable to identify a scientific explanation for it. In HIV patients taking TDF-containing regimens, renal tubular failure can occur in asymptomatic patients with normal eGFR and is linked to electrolyte and micronutrient wasting. The causes of TDF tubulopathy are not particular. As a result, researchers advised conducting a comparison between renal biopsies and Fanconi syndrome parameters when screening for TDF side effects. As TDF side effects have occasionally been found to be permanent, researchers advise considering switching to ABC or TAF instead.

Nishijima et al. [26] study demonstrated a substantial correlation between KTD and cumulative TDF exposure, regardless of current TDF use. This was done by utilizing both the standard definition of KTD, which uses five tubular markers, and alternative definitions, which substitute tubular proteinuria for the tubular marker with the highest or lowest prevalence. It is noteworthy that patients who had previously used TDF but stopped were more likely to present with KTD than patients who had never used TDF if cumulative exposure to TDF exceeded two years. This is true even though patients who had never used TDF were more likely to have risk factors for KTD, such as older age, comorbid conditions, longer time from HIV-1 diagnosis, and longer duration of ART than past TDF users. The findings raise questions regarding whether TDF-associated KTD in patients who stopped using TDF or intended to stop taking it, especially because many patients have switched to taking TAF in resource-rich settings or will do so in the future.

The present data further supports the notion that beta 2 microglobulin is a sensitive marker for TDF tubulopathy among the tubular indicators. NAG is not a sensitive indicator of TDF tubulopathy; however, as evidenced by the lack of a significant difference in the prevalence of high NAG values between the three groups (current TDF users: 25%, previous TDF users: 28%, never TDF users). Inversely correlated with KTD were higher body weight, higher eGFR, higher current CD4 cell count, older age, history of AIDS, hypertension, and longer time from HIV-1 infection diagnosis. Most males in the study were Asian. If these findings apply to females and patients from different ethnic backgrounds, more research is required to validate this.

A systematic review was conducted by Mtisi et al. [27] to assess the renal safety of TDF in Africans and to document the occurrence of kidney illness in HIV-positive Africans on ART including TDF. The study reported statistically significant renal function decline related to TDF use; however, the clinical relevance of this effect was not enough to suggest that TDF should not be continued in ART regimens.

Our research clearly shows that TDF use is linked to a significant decrease in renal function, while the clinical degree of this effect varies. Despite TDF’s effectiveness in reducing viral load and its affordability, the percentage of chronic and end-stage renal disease appears to be increasing over time. As a result, patients continue to have a disproportionately high cumulative risk of suffering from either acute or chronic renal injury or renal tubular disease. Unfortunately, there is no complete evidence for the complete reversibility of the mentioned conditions. Furthermore, these kidney conditions are probably overlooked, identified too late, underreported, and are still completely underappreciated in clinical practice.

Numerous studies indicate that patients on TDF should be routinely monitored. When TDF is used as the first-line drug in a HAART regimen, there is a gap in the application of stringent regulations and monitoring techniques. Our analysis brought attention to the fact that, despite monitoring techniques, there is no proof of renal safety, especially when taken for a prolonged period. Tenofovir is still used extensively in areas with inadequate resources because of the suggestion to adopt the test-and-treat strategy. It is safer to think about TAF or other antiretroviral medication classes. With a safety profile tailored to reduce concomitant risks to long-term health, it requires less monitoring.

## Conclusions

Our research shows that being exposed to TDF-based ART is associated with an increased risk of renal impairment. In conclusion, the renal consequences of TDF remain important, especially as more patients start first-line ART, which includes TDF, globally. Beginning TDF medication put patients with CKD at risk of additional renal function loss, and continued TDF exposure raised the likelihood of an incomplete recovery. Being on long-term TDF-based ART has the potential to cause tubular disease and CKD, where reversal is a risk.

Given that new patients begin treatment with TDF-based regimens in countries with low resources where it is logistically impractical to create screening and monitoring protocols, the continued use of this approach must be examined. Alternatively, sometimes lab results may not show the earliest changes of TDF-induced kidney impairment. We advise introducing alternate pharmacological options as soon as possible. The assessment of serum creatinine and eGFR before the initiation of TDF-containing ART is critical while we implement the HIV test and start method.
